# Supplementary material for: Mild Acquired von Willebrand Syndrome and Cholestasis in Pediatric and Adult Patients with Fontan Circulation
Source: J Clin Med. 2023 Feb 3;12(3):1240. doi: 10.3390/jcm12031240 (PMC9917608; doi:10.3390/jcm12031240)
Supplement: Supplementary file 1 [file jcm-12-01240-s001.zip › jcm-2151123-supplementary.pdf]

Supplemental Figures:

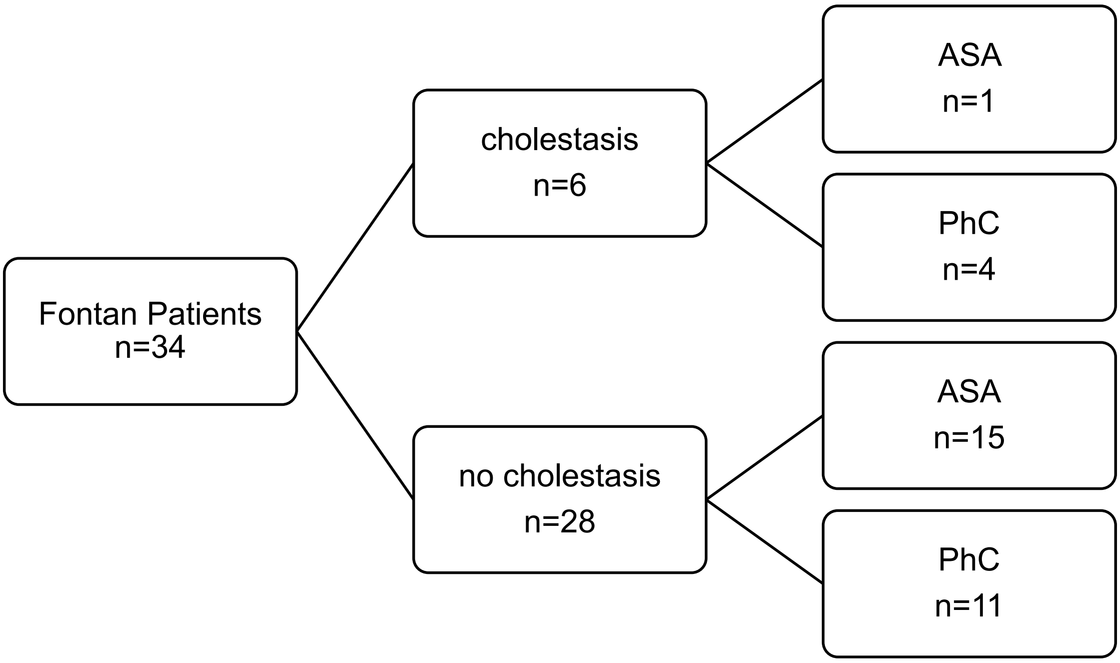

Figure S1: Overview of groups and subcohorts investigated in this study.
Abbreviations: ASA, acetylsalicylic acid; PhC, Phenprocoumon

Supplemental Tables:

Table S1. Unconjugated bile acids (BA) and their glycine (G) or taurine (T) conjugates analyzed in this study design.

Unconjugated Bas	G- conjugated BA	T- conjugated BA
Cholic acid (CA)	GCA	TCA
Chenodeoxycholic acid (CDCA)	GCDCA	TCDCA
Deoxycholic acid (DCA)	GDCA	TDCA
Lithocholic acid (LCA)	GLCA	TLCA
Ursodeoxycholic acid (UDCA)	GUDCA	TUDCA

Table S2: Descriptive data and results of the Mann-Whitney U tests, comparing the relative bile acid (BA) values between cholestatic and non-cholestatic Fontan patients.

	Compared subcohorts	n	Mean	IQR	p-value
**GCA**	cholestasis	6	0.323	0.317-0.382	**<0.001**
	no cholestasis	28	0.148	0.093-0.203	
**GCDCA**	cholestasis	6	0.331	0.258-0.386	0.364
	no cholestasis	28	0.397	0.228-0.480	
**GLCA**	cholestasis	6	0.018	0.011-0.035	0.514
	no cholestasis	28	0.051	0.001-0.175	
**GDCA**	cholestasis	6	0.759	0.605-1.165	0.439
	no cholestasis	28	0.561	0.371-1.188	
**GUDCA**	cholestasis	6	0.206	0.187-0.315	0.220
	no cholestasis	28	0.292	0.197-1.040	
**TCA**	cholestasis	6	0.063	0.042-0.096	**0.011**
	no cholestasis	28	0.022	0.009-0.049	
**TCDCA**	cholestasis	6	0.782	0.591-1.039	0.5221
	no cholestasis	28	0.675	0.448-0.919	
**TLCA**	cholestasis	6	0.000	0.000-0.000	**0.012**
	no cholestasis	28	0.000	0.000-0.000	
**TDCA**	cholestasis	6	0.162	0.097-0.531	0.947
	no cholestasis	28	0.177	0.101-0.250	
**TUDCA**	cholestasis	6	0.002	0.002-0.003	**0.010**
	no cholestasis	28	0.000	0.000-0.001	
**CA**	cholestasis	6	0.063	0.023-0.198	0.310
	no cholestasis	28	0.036	0.000-0.171	
**CDCA**	cholestasis	6	0.178	0.095-0.284	0.121
	no cholestasis	28	0.287	0.146-0.736	

**LCA**	cholestasis	6	0.019	0.010-0.037	0.431
	no cholestasis	28	0.034	0.001-0.096	
**DCA**	cholestasis	6	0.118	0.068-0.159	0.172
	no cholestasis	28	0.279	0.078-0.895	
**UDCA**	cholestasis	6	0.003	0.002-0.006	**< 0.001**
	no cholestasis	28	0.026	0.010-0.097	

Significant differences are shown in bold.
Orange = glycine conjugated BA, green = taurine conjugated BA, blue = unconjugated BA.
